# Genetic programming based models in plant tissue culture: An addendum to traditional statistical approach

**DOI:** 10.1371/journal.pcbi.1005976

**Published:** 2018-02-27

**Authors:** Meenu R. Mridula, Ashalatha S. Nair, K. Satheesh Kumar

**Affiliations:** 1 Department of Botany, University of Kerala, Kariavattom, Thiruvananthapuram, Kerala, India; 2 Department of Future Studies, University of Kerala, Kariavattom, Thiruvananthapuram, Kerala, India; Laboratoire Jean Kuntzmann, FRANCE

## Abstract

In this paper, we compared the efficacy of observation based modeling approach using a genetic algorithm with the regular statistical analysis as an alternative methodology in plant research. Preliminary experimental data on *in vitro* rooting was taken for this study with an aim to understand the effect of charcoal and naphthalene acetic acid (NAA) on successful rooting and also to optimize the two variables for maximum result. Observation-based modelling, as well as traditional approach, could identify NAA as a critical factor in rooting of the plantlets under the experimental conditions employed. Symbolic regression analysis using the software deployed here optimised the treatments studied and was successful in identifying the complex non-linear interaction among the variables, with minimalistic preliminary data. The presence of charcoal in the culture medium has a significant impact on root generation by reducing basal callus mass formation. Such an approach is advantageous for establishing *in vitro* culture protocols as these models will have significant potential for saving time and expenditure in plant tissue culture laboratories, and it further reduces the need for specialised background.

## Author summary

Trials to find out the best combination of factors that contribute to the desired response takes up the chunk of time and resources in any plant tissue culture laboratory. The output of such experiments is analysed statistically to come to a conclusion. However, without prior statistical modifications, the results could be misleading. Recent reports from several labs point out the use of artificial neural networks to circumvent this. We have chosen to use a computational process that can predict the best combination of factors for the desired response after randomly testing the higher and lower limit of the factors with experiments. The magnitude of the desired response can be presumed at any concentration within this range using the models generated by symbolic regression. The procedure provides both optimum model function as well as the optimum variable values in the model. The variable sensitivity and percentage response add depth to the information thus obtained. The study indicated that these models would have significant potential for saving time and expenditure in plant tissue culture laboratories for the commercial establishment of *in vitro* protocols.

## Introduction

Relatively more straightforward and efficient empirical modeling techniques based on input-output models are gaining popularity to conventional statistical methods across various disciplines [1]. This surge is due to its relative ease of use and understanding. Genetic programming (GP) is an approach which uses the concept of biological evolution to handle a problem with many fluctuating variables. Computational optimisation techniques have recently debuted in plant tissue culture research as studied in neural networks models [2]. Symbolic regression was one of the earliest applications of GP and continues to be widely considered [3]. A broad array of scientific fields like Biology, Chemistry, Environmental Science, N*e*urology and Psychology reports the use of symbolic regression [4–9]. However, plant tissue culture data has not yet been analysed using symbolic regression. The data generated from plant tissue culture experiments includes continuous, count, binomial or multinomial and predominantly the information is validated using analysis of variance method (ANOVA) [2,10]. ANOVA is adequate for normally distributed continuous data; but without prior manipulation, it is erroneous to analyze count, binomial or multinomial data [11]. Neuro-fuzzy logic is the standard practice by which computational modeling is achieved in plant tissue culture [2,12]. In this context, genetic algorithm based symbolic regression remains unevaluated. Unlike conventional regression analysis which optimises parameters for a pre-defined model, symbolic regression avoids imposing any *apriori* assumptions. In generalised linear model (GLM) regression, the dependent variable is represented as linear combination of the given set of basic functions and optimise the coefficients to fit the data. However, symbolic regression searches for both a set of basic functions and coefficients. The added value of symbolic regression, compared to GLM, lies in its ability to quickly and accurately find an optimal set of basic functions [13,14]. The algorithm infers the model from the data by combining variables and mathematical operators and generates an empirical formula which is a mathematical equation that predicts observed results derived from conducted experiments. GP combines previous equations and forms new ones. Thus it produces models with interpretable structure, relating to input and output variables from a data set without pre-processing and identifying critical parameters and hence shed insight into the underlying processes involved in a given system [15]. Symbolic regression can recognise and model complex non-linear relationships between the inputs and outputs of biological processes even in the presence of disturbances and potential for parallel processing. The preliminary data generated from experiments during rooting of *in vitro* regenerated plantlets in *Wrightia tinctoria* was employed to study the utility of symbolic regression to analyze plant tissue culture data. The effect of two variables - NAA and charcoal on root proliferation was considered. The datasets were subjected to usual statistical analysis as well as observation based modeling via symbolic regression. Moreover, we aimed to optimise the process by examining the influencing factors. We propound the use of symbolic regression-based model prediction as an addendum to data analysis method for plant tissue culture experiments.

## Materials and methods

### Culture conditions

The genetic variability was kept minimum by using a single field grown ortet, thus minimising statistical errors [16]. Nodal regions derived from the fresh flushes of growth from the ortet, two weeks after lopping one major branch served as the explants [17]. The nodal explants were conditioned over a period of 4 months (subculture/four weeks) on MS medium (1962) [18], pH 5.8 and 2 μM each of BAP and NAA for shoot multiplication. For rooting experiments individual shoots were transferred on MS medium containing 2 μM BAP with NAA (2, 4 and 6 μM, respectively) and charcoal (0.01, 0.03, 0.05, 0.07, 0.09 and 0.11%, respectively) in 250 ml culture flasks in 50 ml of sterilized medium (pH 5.8). The cultures were maintained at 25±2°C in a culture room with 40 μmolm^−2^ s^−1^ irradiances and a photoperiod of 8 hrs with 55±5% of relative humidity.

### Experimental design

The plant tissue culture database, containing 21 conditions, followed a factorial design for two variables- concentration of NAA (2, 4 and 6 μM) and charcoal (0,0.01, 0.03,0.05,0.07,0.09 and 0.11%) in the medium. Each treatment consisted of 5-7 explants in a culture flask with three replicates. The subculture was done at the end of four weeks and five parameters were recorded to analyze the effects of the variables on rooting such as basal callus diameter (mm) (BC), the percentage of shoots rooted (R), the length of the longest root (cm) (RL), the number of roots (NR) and the number of lateral roots (NLR) (S1).

### Statistical analysis

All experiments were conducted using Randomised Block Design (RBD). Continuous data were analysed using multiple linear regression in R and posthoc comparisons of pairs performed by Tukey's test (p>0.05). Count data were analysed using Poisson regression model. Pearson's Chi-squared test for count data was employed to access statistical significance of the variables.

### Symbolic regression

Each of the observed parameters is modeled as a function of NAA and charcoal concentrations using symbolic regression and GLM for comparison. To obtain a global optimum, we have also modelled the combination (R+RL+NR+NRL-BC) by taking rooting factors together after normalisation by employing both GLM and symbolic regression. The optimum model for each case was generated by genetic programming based symbolic regression using the software package Eureqa (Version 0.98 beta) with 50% of the data randomly selected as training data, and 3-fold cross-validated with randomly selected 25% of the remaining data [19–21]. Corresponding to each symbolic regression model of the data partition, we have also obtained generalised linear model by including x, y, xy, 1/x, 1/y, sin(x), cos(x), sin(y), cos(y), xy sin(x), xy cos(x), xy sin(y), xy cos(y) into the set of basic functions and cross-validated similarly. The remaining 25% the data was used for testing and reporting error [19–21]. The Target expression used to generate the regression model was the minimal equation z = f(x, y) where ‘x' corresponds to NAA concentration and ‘y' corresponds to charcoal concentrations, and ‘z' represents each of the five observed parameters and their combination. The models were based on the primary and trigonometric building blocks, with the R^2^ goodness of fit as the error metric [22,23]. Root Mean Squared Error (RMSE) was calculated for the test data sets. Sensitivity represents the relative impact of the variable on the parameter studied within this model and was calculated by the local method using the partial derivatives [24]. Given a model equation of the form z = f(x, y), the influence metrics of x on z was;

$$\mathrm{Sensitivity}=\bar{\left|\frac{\partial z}{\partial x}\right|}.\frac{\sigma (x)}{\sigma (z)},\;\mathrm{evaluated}\;\mathrm{at}\;\mathrm{all}\;\mathrm{input}\;\mathrm{data}\;\mathrm{points};$$

The percentage positive was calculated as percentage of data points where $\frac{\sigma (x)}{\sigma (z)}>0$ and percentage negative was calculated as percentage of data points where $\frac{\sigma (x)}{\sigma (z)}<0$; where $\frac{\partial z}{\partial x}$ was the partial derivative of z with respect to x, σ(x) was the standard deviation of x in the input data, σ(z) was the standard deviation of z, |x| denoted the absolute value of x, and $\bar{x}$ denoted the mean of x [25]. The ‘fmin' function in MATLAB (R2012b) was used to obtain the maximum value of each of these functions.

## Results and discussion

**Fig 1:**
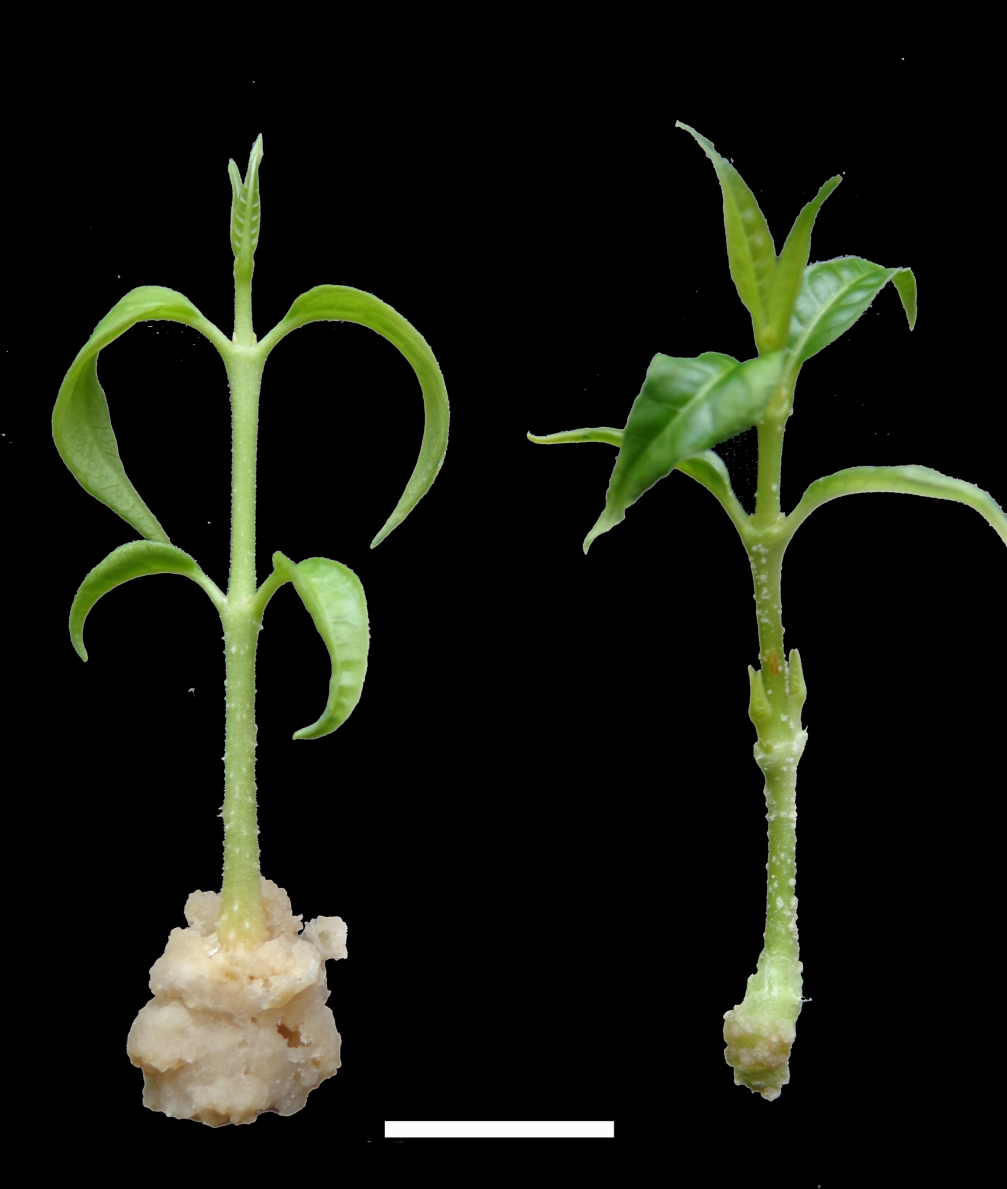
*In vitro* plants of *W*. *tinctoria-* Shoot inoculated in the absence of charcoal showing profuse basal callusing (left); against shoot inoculated in the presence of charcoal showing reduced/no basal callus mass (right).

**Fig 2:**
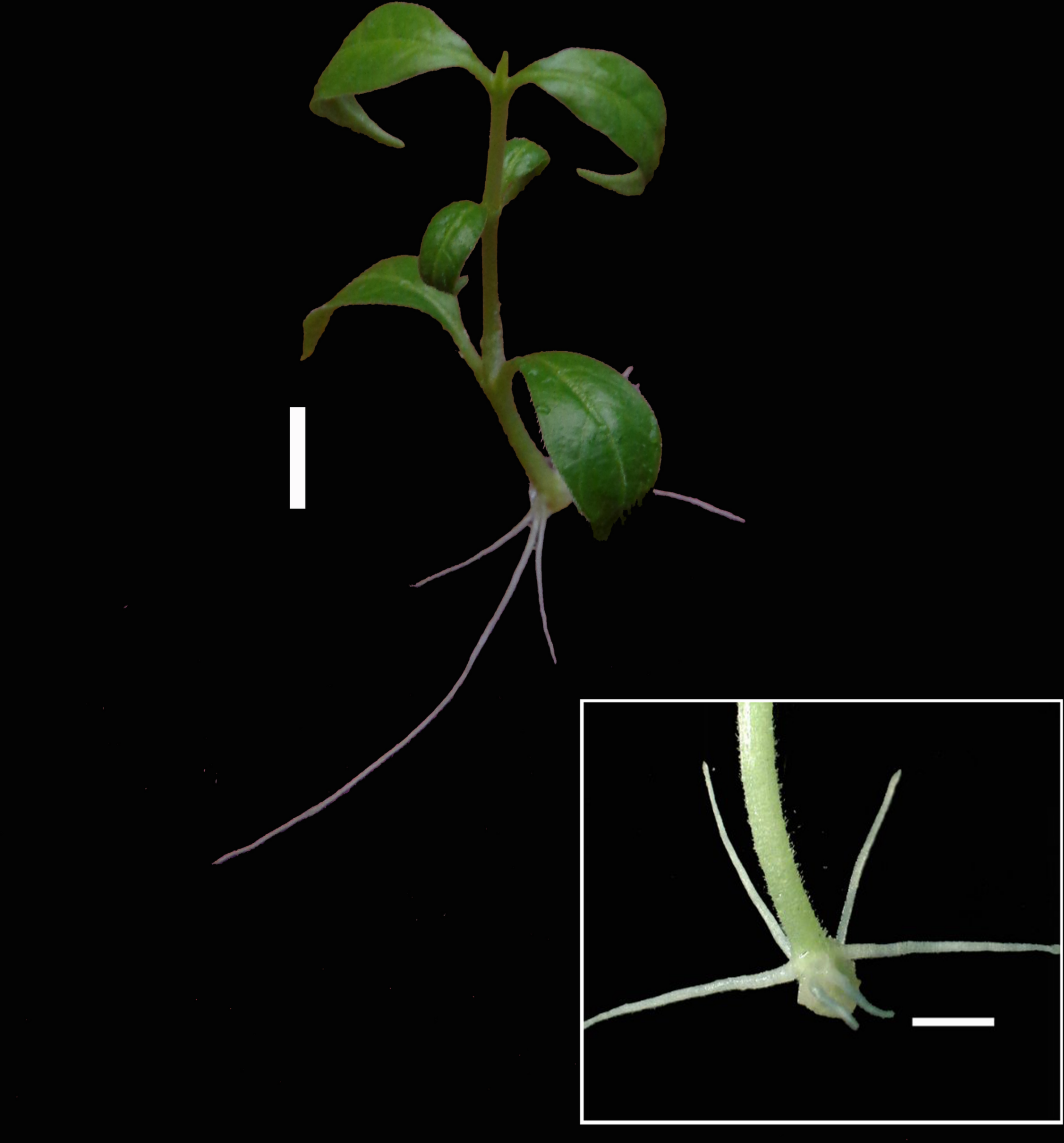
Maximum rooting observed in the shoots inoculated on MS medium supplemented with 4 μM NAA and 0.07% charcoal.

The average values obtained for the five growth parameters observed during the study were given as the basal callus diameter (Table 1), the percentage of shoots rooted (Table 2), the length of longest roots (Table 3) and the number of roots and the number of lateral roots (Table 4). The miniscule alphabets within a column indicated the significant influence of charcoal and majuscule alphabets in the row represented the significant interaction of NAA. The shoots inoculated on MS medium with 0% charcoal (control) showed maximum basal callus formation (Fig 1). The shoots inoculated on MS medium supplemented with 4μM NAA and 0.07% charcoal showed the maximum percentage of rooting (Fig 2).

**Table 1:** Mean of basal callus diameter after four weeks of culture under the conditions studied. Means followed by similar letters; a, b and c (within each column) and A and B (in each row) are not significantly different by Tukey's multirange test (p>0.05).

Basal callus Diameter (mm)	Charcoal	NAA		
		2	4	6
	0	10.06 c A	11.40 d B	11.33 c B
	0.01	2.25 b AB	2.40 c B	2. b A
	0.03	1.61 a A	2.38 c B	1.43 a A
	0.05	1.57 a A	1.54 b A	1.32 a A
	0.07	1.32 a A	1.36 ab A	1.22 a A
	0.09	1.34 a A	1.12 ab A	1.27 a A
	0.1	1.44 a A	1.28 a A	1.24 a A

**Table 2:** Mean of the percentage of shoots rooted after four weeks of culture under the conditions studied. Means followed by similar letters; a, b and c (within each column) and A Band C (in each row) are not significantly different by Tukey's multirange test (p>0.05).

Percentage of shoots rooted	Charcoal	NAA		
		2	4	6
	0	0 a A	0 a A	0 a A
	0.01	0 a A	0 a A	0 a A
	0.03	0 a A	10.76 a B	12.96 b B
	0.05	43.9 c B	47.6 c B	12.8 b A
	0.07	39.93 c B	50 c C	28 c A
	0.09	27.43 b B	31.7 b B	9.4 ab A
	0.1	28.3 b B	23.23 b B	0 a A

**Table 3:** Mean of the length of longest roots after four weeks of culture under the conditions studied. Means followed by similar letters; a, b and c (within each column) and A and B (in each row) are not significantly different by Tukey's multirange test (p>0.05).

Length of longest roots (mm)	Charcoal	NAA		
		2	4	6
	0	0 a A	0 a A	0 a A
	0.01	0 a A	0 a A	0 a A
	0.03	0 a A	7.88 b B	1.72 a A
	0.05	26.4 c C	17.94 c B	6.9 ab A
	0.07	41.78 d B	41.67 d B	14.4 b A
	0.09	18.17 b B	10.1 b A	6.8 a A
	0.1	7.38 a B	3.13 ab AB	0 a A

**Table 4:** Mean of the number of roots and number of lateral roots after four weeks of culture under the conditions studied. The row and the column variables are statistically significantly associated at p>0.05 (Number of roots) and p>0.001(Number of lateral roots), by Pearson's Chi-squared Test for Count Data.

Number of Roots	Charcoal	NAA		
		2	4	6
	0	0	0	0
	0.01	0	0	0
	0.03	0	0.4	0.6
	0.05	5	2.4	1.2
	0.07	1.5	1.4	0.8
	0.09	1	1.13	0.8
	0.1	0.8	0.6	0
Number of lateral Roots	0	0	0	0
	0.01	0	0	0
	0.03	0	0	0
	0.05	0.4	0.43	0
	0.07	5	0.6	1.1
	0.09	0.6	2.7	2.2
	0.1	0	1.96	0

**Fig 3:**
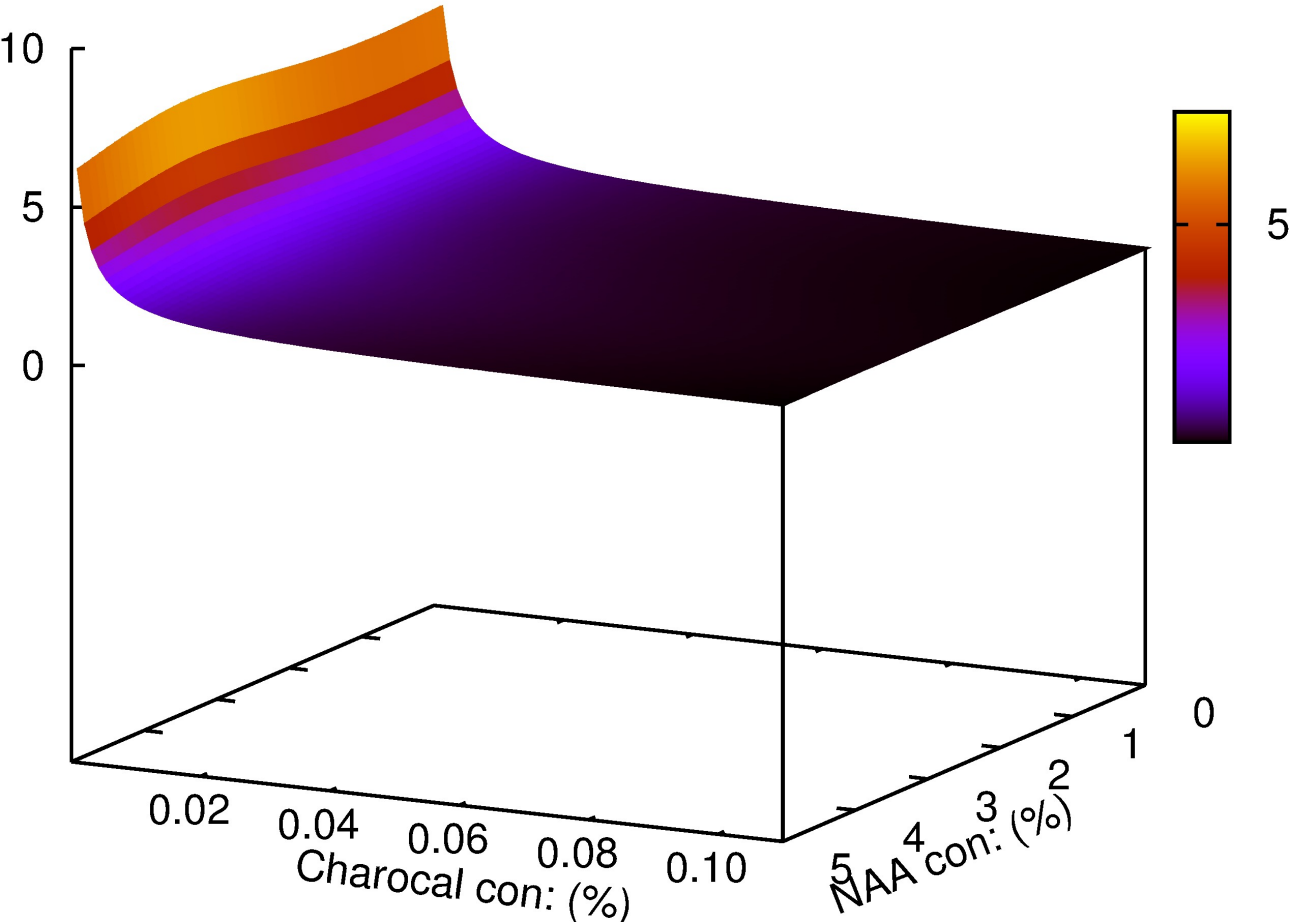
The plot generated for parameters predicted by symbolic regression model for *W*. *tinctoria* plantlets as a function of NAA and charcoal concentration showing basal calli diameter (mm). The cyan coloured dot indicates the optimum concentration predicted by the model function.

**Fig 4:**
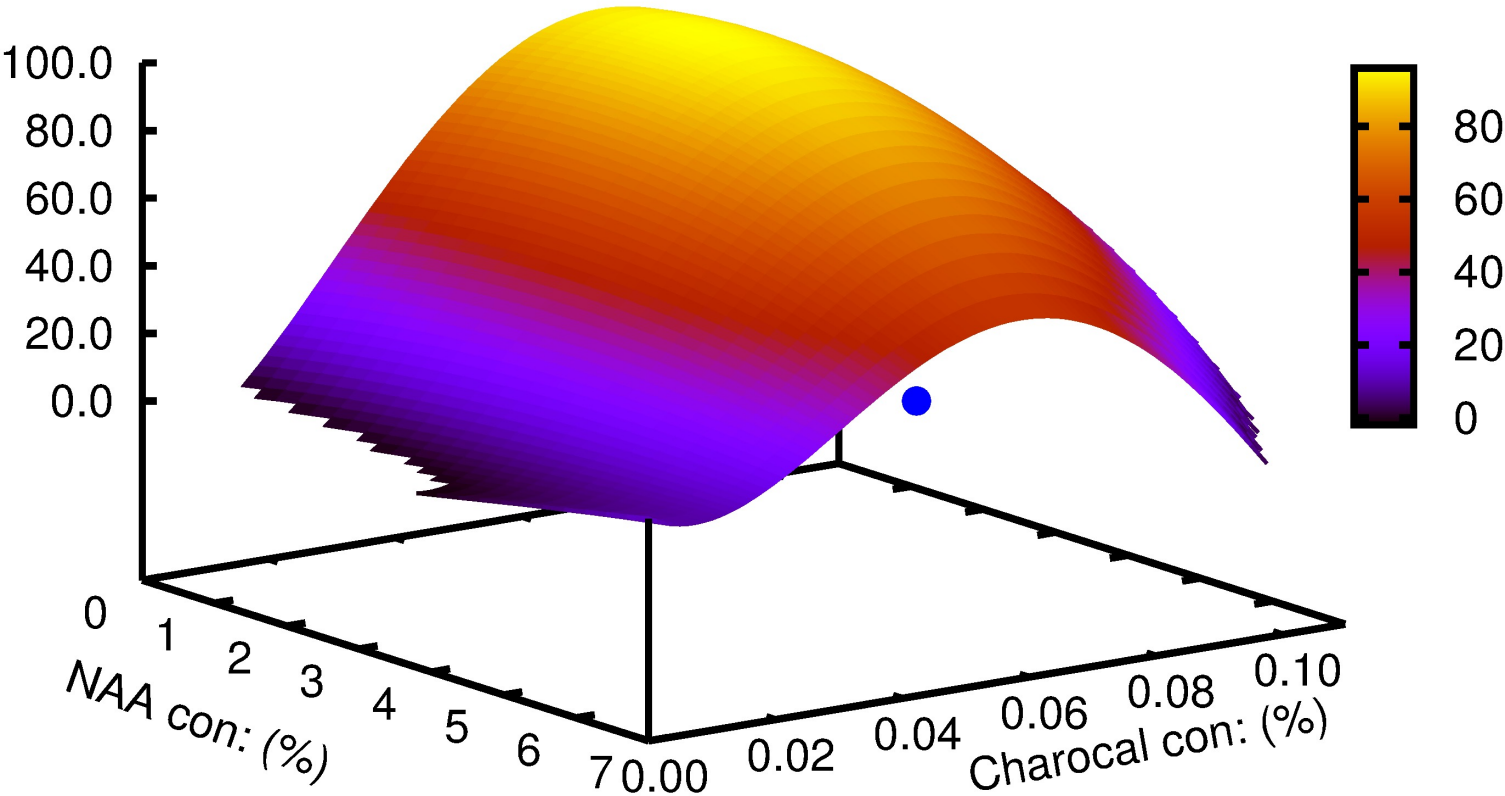
The plot generated for parameters predicted by symbolic regression model for *W*. *tinctoria* plantlets as a function of NAA and charcoal concentration showing rooting percentage. The cyan coloured dot indicates the optimum concentration predicted by the model function.

**Fig 5:**
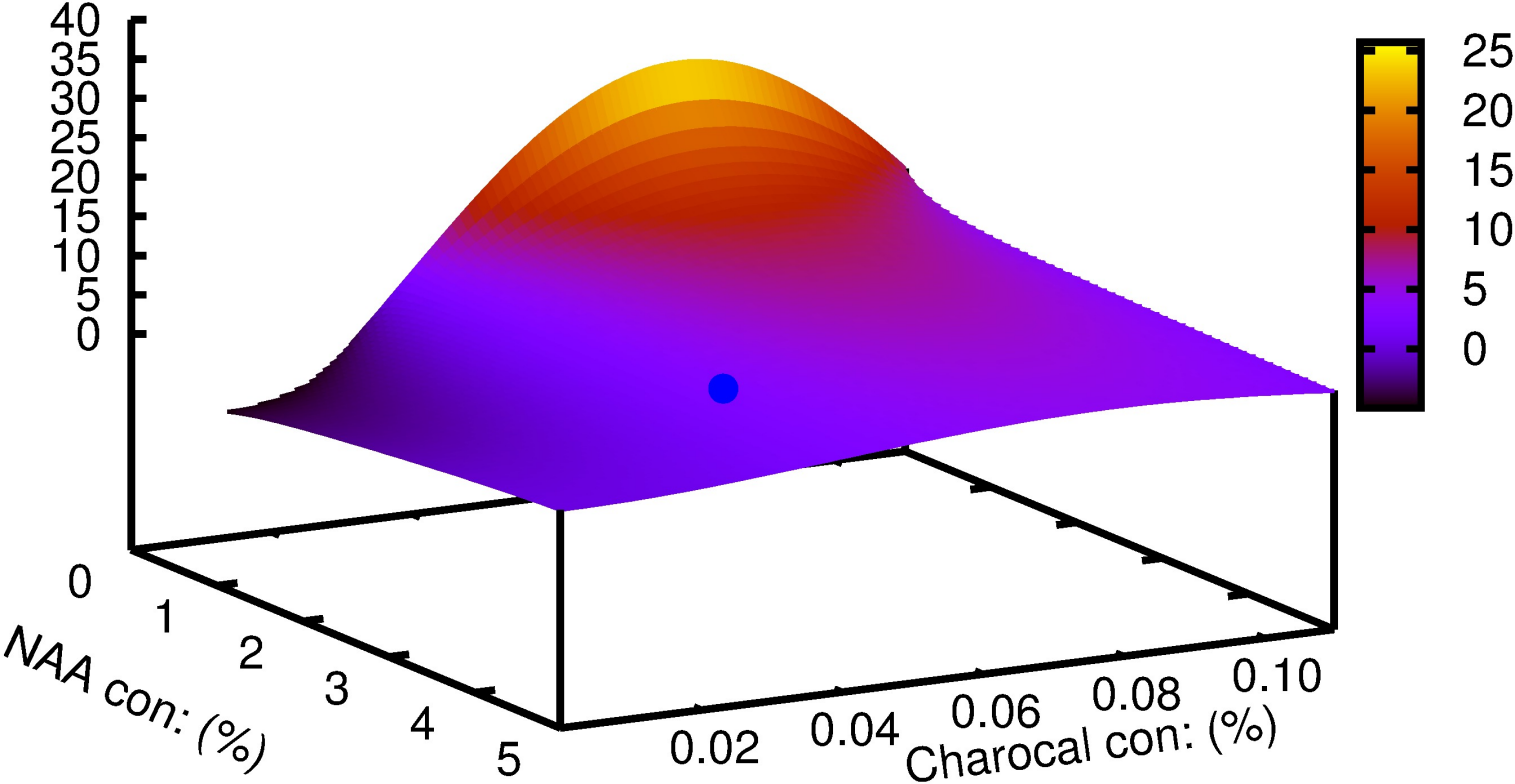
The plot generated for parameters predicted by symbolic regression model for *W*. *tinctoria* plantlets as a function of NAA and charcoal concentration showing the length of the longest roots (mm). The cyan coloured dot indicates the optimum concentration predicted by the model function.

**Fig 6:**
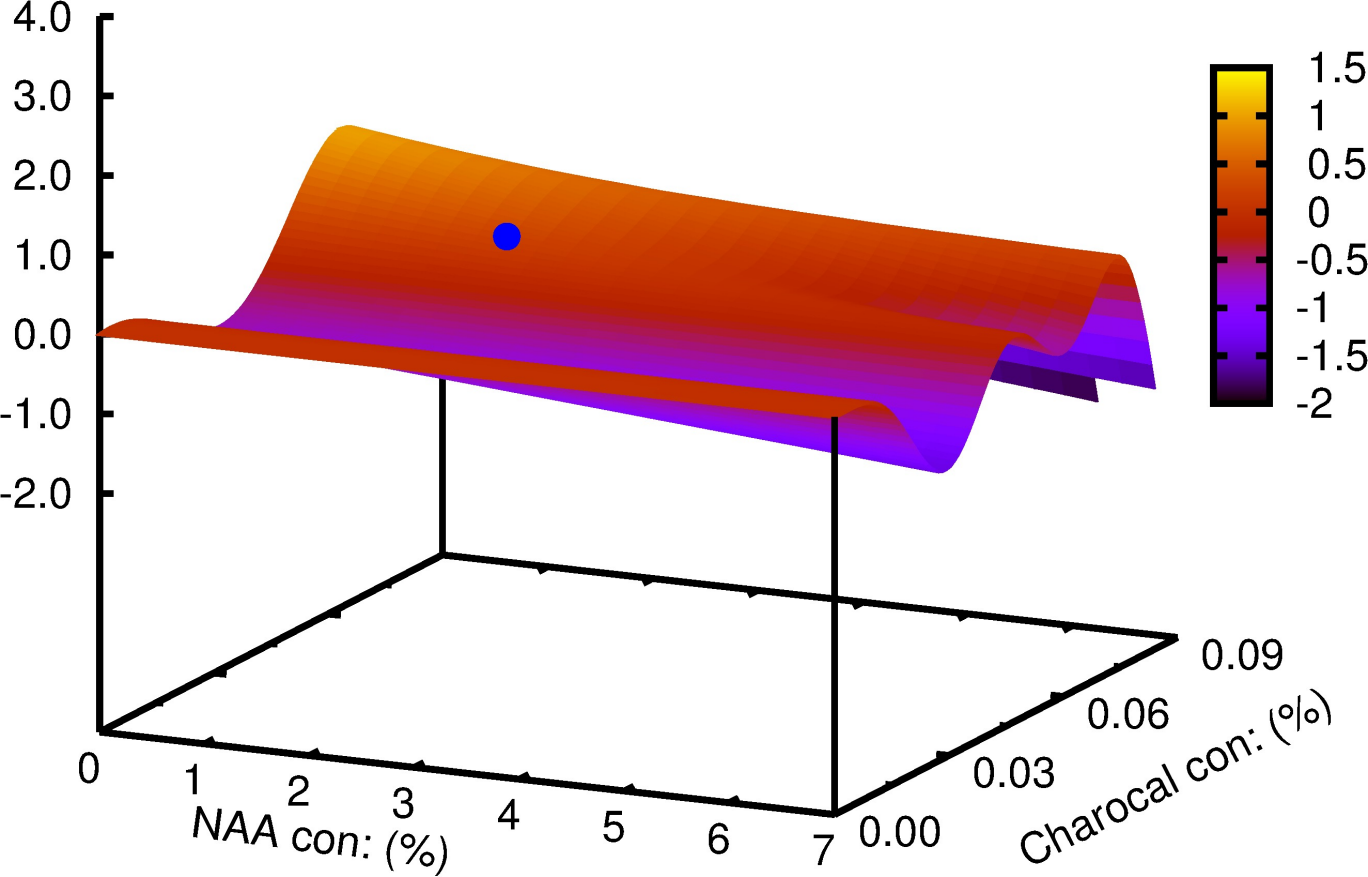
The plot generated for parameters predicted by symbolic regression model for *W*. *tinctoria* plantlets as a function of NAA and charcoal concentration showing the number of roots. The cyan coloured dot indicates the optimum concentration predicted by the model function.

**Fig 7:**
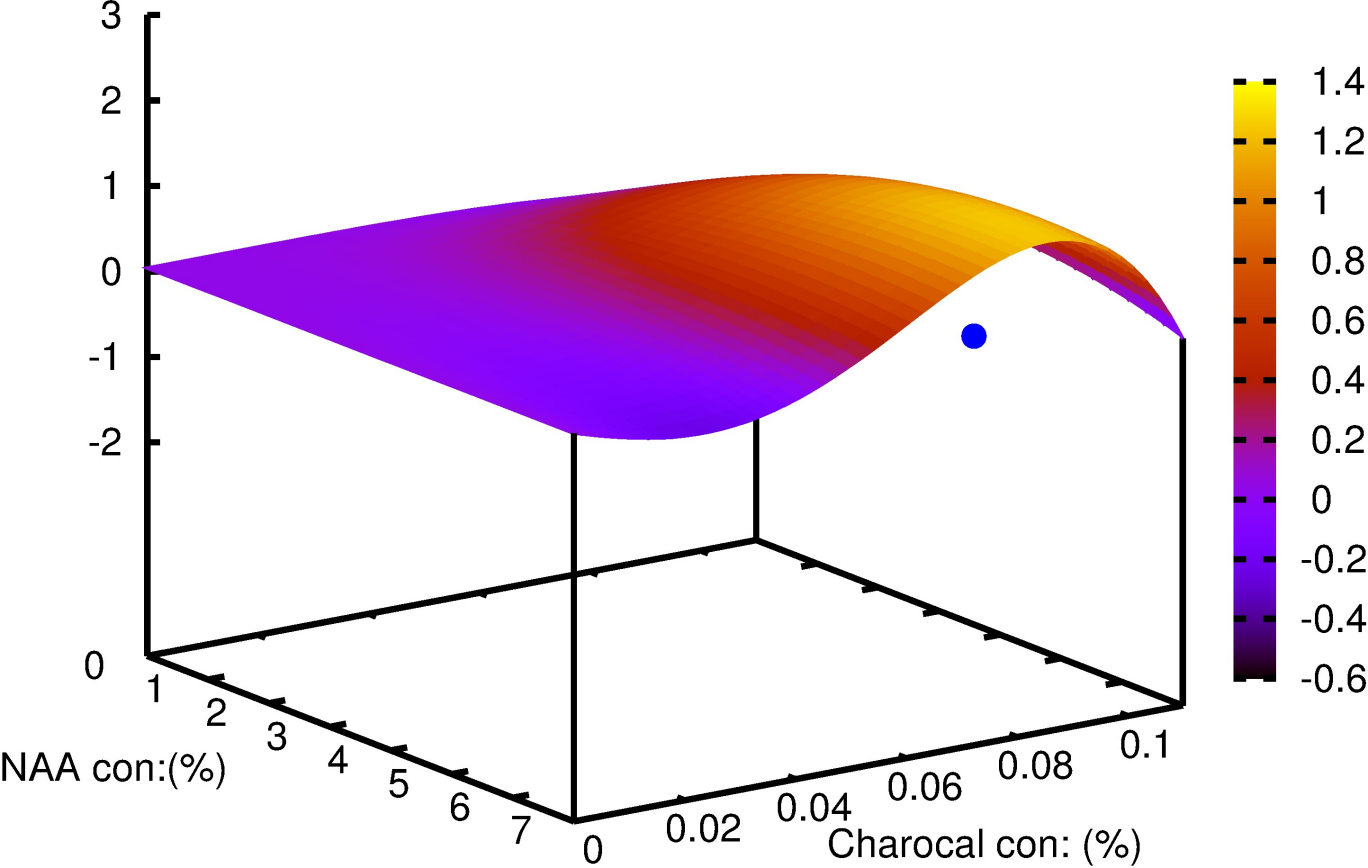
The plot generated for parameters predicted by symbolic regression model for *W*. *tinctoria* plantlets as a function of NAA and charcoal concentration showing the number of lateral roots. The cyan coloured dot indicates the optimum concentration predicted by the model function.

**Fig 8:**
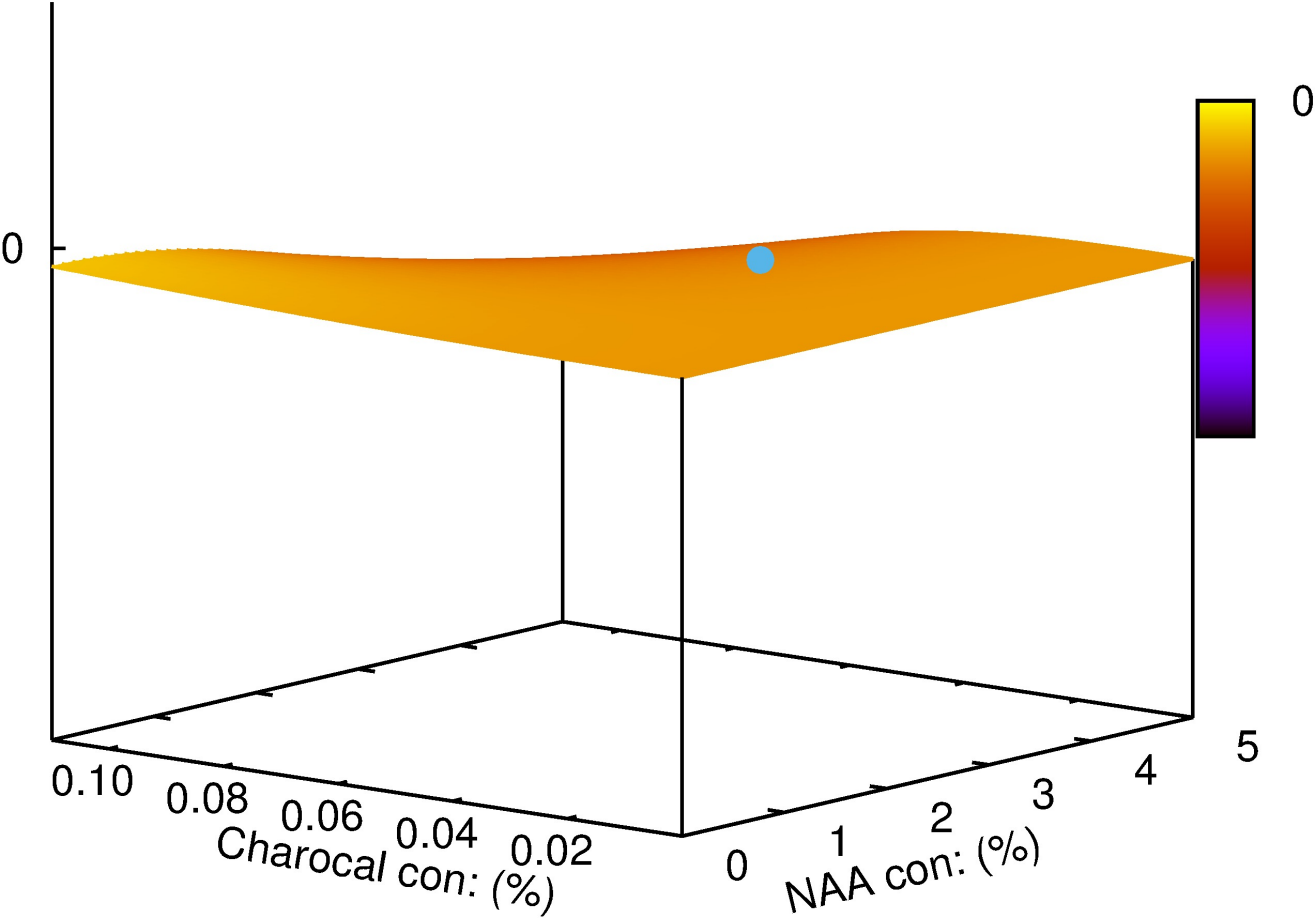
The plot generated for the combination of all parameters studied, by symbolic regression model for *W*. *tinctoria* plantlets as a function of NAA and charcoal concentration. The cyan coloured dot indicates the optimum concentration predicted by the model function.

Multiple linear regression demonstrated a significant effect of NAA and its interaction with charcoal on basal callus (p>0.001), the percentage of shoots rooted (p>0.05) and root length (p>0.01) (Tables 1–3). The individualistic effect of NAA for the number of roots and lateral roots were found to be significant at p>0.05 and p>0.001 respectively (Table 4). The interaction of NAA and charcoal was not significant for the same parameters studied. Mathematical functions were successfully developed using symbolic regression to understand the correlation between the two variables for each of the parameters considered and is contrasted with those obtained by traditional regression models (Table 5). To analyze the effect of each of the variables on the parameter studied; variable sensitivity measures were calculated along with its percentage impact. Its sensitivity denoted the relative impact within this model that a variable has on the target variable. The individualistic effect of the two input variables on the output parameter was pointed out as percentage positive or negative of that input variable (Table 6). For the parameter basal calli diameter, the percentage positive value for variable ‘y' was zero. In other words, there was zero percent chance of basal calli mass increasing with increasing concentration of charcoal; or that basal calli mass decrease with increasing concentration of charcoal (Fig 3). The model predicted that increase in charcoal concentration had a consequent increase in root length and root number in 50% of all the trials while the same promoted rooting percentage and lateral root number in 75% of the trials. Root number and root length decreased with increasing concentration of NAA in 100% of the trials. Rooting percentage and lateral root numbers increased with increasing NAA concentration in 50% of all the trials. The function obtained and the 3D plots thus generated could be used to predict the combinations of input variables giving optimum results. The best response for rooting percentage was predicted at 3.7 μM NAA and 0.08% charcoal (Fig 4). The root length showed a non-linear pattern, and the highest value for its function was estimated with 2.8 μM NAA and 0.05% charcoal (Fig 5). The maximum root number was determined for 1.7 μM NAA and 0.06% charcoal (Fig 6). The maximum value of the function generated for lateral root number was with 6.3 μM NAA and 0.08% charcoal (Fig 7). The global optimum modelled upon the combination (R+RL+NR+NRL-BC) indicated the results as 2.44 μM NAA and 0.03% charcoal (Fig 8).

**Table 5:** Functions and error matrix obtained through symbolic regression and multiple linear regression.

Parameter	Symbolic regression			Linear regression		
	Function	R^2^	RMSE	Function	R^2^	RMSE
Basal callus diameter (BC)	1.17 + 0.12/(0.01 + y)	0.98	0.09	739.5 x – 51540 y + 73390 Sin(y) – 0.56 Sin(x) -29450 Cos(y) – 1190 xy + 1673 x Sin(y) – 14130 y Sin(y) +739.3 x Cos(y) -21970 y Cos(y) + 0.7174 y Sin(x) -350.2 xy Sin(y) -487.8 xy Cos(y) +294660	0.98	0.56
Percentage of shoots rooted (R)	4.43 x + 4738572.57 y^4^ + 88353.22 y^2^ - 18.33 – 1270650.34 y^3^ – 21.85 y x^2^	0.73	0.94	70990 x + 619700 y -904900 Sin(y) + 0.294 Sin (x) + 278000 Cos(y) -157200 xy + 229300 x Sin(y) + 137300 y Sin (y) -70990 x Cos(y) + 285300 y Cos(y) -27.01 y Sin(x) -34940 x y Sin (y) -72090 x y Cos(y) -278000	0.60	1.11
Length of longest roots (mm) (RL)	2.28 -22.58 cos(40.39 y)/(1.25 x + x cos(5.06 y) + sin(1.84 - x))	0.75	1.28	46840 x + 530000 y -768300 Sin(y) -1.7 Sin(x) + 263300 Cos (y) -98130 xy + 142600 x Sin(y) + 129200 y Sin(y) + 129200 y Sin(y) -46840 + 238500 y Cos(y) -4.134 y Sin(x) -22990 x y Sin (y) -44490 x y Cos(y) -263300	0.85	7.85
Number of roots (NR)	14.83 y^2^ + 14.78 y^2^ sin(10.50 + 6375y) – 4.80 y – 1.92 x y -19.2 y sin(5.5 + 88.86y) – 1.95 x y sin(6.3-183.03y)	0.82	0.76	5984 x + 56560 y -83160 Sin(y) + 0.09042 Sin(x) + 22980 Cos(y) -14110 xy + 20670 x Sin (y) + 11420 y Sin(y) -5984 x Cos (y) + 26590 y Cos(y) -0.3519 y Sin (x) -2959 x y SIn (y) -6560 x y Cos(y) -22980	0.63	1.03
Number of lateral roots (NRL)	0.04 + 2208.78 x y^3^ - 3559.64 y^4^ – 0.39 y x^2^ – 17758.21 x y^4^	0.96	0.02	7025 x + 56710 y -82060 Sin(y) + 0.467 Sin (x) + 28560 Cos(y) -14700 x y + 21340 x Sin(y) + 13970 y Sin(y) -7025 x Cos(y) + 25370 y Cos(y) -2.28 y Sin(x)-3443 xy Sin(y)-6652 xy Cos (y) -28560	0.68	1.40
Normalised (R+RL+NR+NRL-BC)	1.24 sin(3.24 + 3.42 x^3^y^2^sin(y) - 3.77y^2^cos(x))	0.80	0.92	8604 x + 15570y – 19520 xy – 2.067 sin(x) – 22750 sin(y) +6988 cos(y) + 3453 y sin(x) -8606 x cos(y)– 4249 xy sin(y) -9014 xy cos(y) - 6988	0.78	1.99

The variable x represents NAA concentration and y represents charcoal concentration

**Table 6:** Variable sensitivity values of the parameters studied through symbolic regression.

Parameters	Sensitivity		Percentage positive		Percentage negative	
	x	y	x	y	x	y
Basal calli diameter (mm)	-	1.65	-	0	-	100
Percentage of shoots rooted	0.7	1.78	50	75	50	25
Length of longest roots (mm)	0.3	1.4	0	50	100	50
Number of roots	0.21	1.26	0	50	100	50
Number of lateral roots	0.18	1.94	50	75	50	25
Normalised	0.13	1.64	50	75	50	25

The conclusion obtained by traditional statistics suggested that charcoal had a positive and stimulatory effect in rooting of shoots by reducing basal callus (Table 1). Percentage of shoots rooted and root length showed a significant impact with the combination of NAA and charcoal (Tables 2 and 3). In the present study, NAA has a significant effect on rooting as shown by the number of roots and lateral roots (Table 4). Similar results were reported in *Acacia leucophloea* and *Cinnamomum verum* [26, 27]. With traditional statistics, we were not able to estimate the combination/s of both variables in producing the best results or able to identify the relative impact of a particular variable on the output parameter. Modeling of plant tissue culture data is practised using regression analysis where first an initial function is approximated and the data fitted to that function to obtain the optimum parameters [11,28,29]. In this procedure even when one gets the optimum parameter values, the model prediction was limited by the probable wrong selection of the model function. In contrast, symbolic regression procedures work simultaneously on model specification problem and the problem of fitting coefficients [30]. Thus it provides both optimum model function as well as the optimum variable values in the model. The simple relations derived from GP were more accessible to analyze the relationships between the input and output variables [31]. Observation-based predictive models using GP identified that the individualistic effect of charcoal was significant in all the output parameters. A previous investigation suggested basal callus mass formation as one of the primary constraints in the culture of this tree species [32]. In the present study, charcoal has a positive and stimulatory effect in rooting by reducing basal callus formation in shoots. For each of the functions, generated values can be obtained by increasing /decreasing the variables by a unit. After randomly testing the higher and lower limit of the additives with experiments, the magnitude of the observed parameters can be presumed at any concentration of the additives within this range using the models generated. It can be extended to analyze synergistic interactions between two parameters by testing whether increasing both variables by a unit, gives a higher or a lower value than the sum of the values obtained by increasing each individually by a unit. The basic requirement for any empirical model includes interpretability, robustness and reliability [33]. Symbolic regression gave comparably lesser RMSE values in comparison to multiple linear regression, thus adding validity to its use. In plant tissue culture obtaining an optimum model is crucial when one needs to find the optimum experimental parameters for large-scale production. The procedure adopted in the work can also be extended to similar experiments as it is general and computationally efficient. The analysis predicted the optimum concentration of medium for micropropagation of the selected tree species from the model plots derived from the preliminary experimental data. The study indicated that these models would have significant potential for saving time and expenditure in plant tissue culture laboratories for the commercial establishment of *in vitro* protocols in tree species.

## Supporting information

S1 Data The minimal data set used for the analysis. Legends NAA, CH, BC, R, RL, NR and NRL represent the concentration of NAA, concentration of charcoal, basal callus diameter, percentage of shoots rooted, length of longest roots, number of roots and number of lateral roots, respectively.
(CSV)
